# Supplementary material for: Evaluating and Enhancing Japanese Large Language Models for Genetic Counseling Support: Comparative Study of Domain Adaptation and the Development of an Expert-Evaluated Dataset
Source: JMIR Med Inform. 2025 Jan 16;13:e65047. doi: 10.2196/65047 (PMC11783024; doi:10.2196/65047)
Supplement: Multimedia Appendix 2 [file medinform_v13i1e65047_app2.pdf]

- Web
  - https://www.jsgc.jp/faq.html
  - https://genmed.kyushu-u.ac.jp/personnel/qa.html
  - https://genmed.kyushu-u.ac.jp/genetic/
  - https://genmed.kyushu-u.ac.jp/genetic/faq.html
  - https://www.fujita-hu.ac.jp/~genome/gc/qa/index.html
  - https://www.ningen-dock.jp/ningendock/pdf/DOCK-idensiQA.pdf
  - https://www.harefukutsuu-hae.jp/familytest/GeneticCounseling/
  - https://www.scchr.jp/department/genomic_medicine/index/genetic_couseling.html
  - https://www.jpeds.or.jp/uploads/files/20200225_iden_qa.pdf
  - https://www2.hosp.med.tottori-u.ac.jp/departments/medical/gene-diagnosis/sp/faq.html

- Books and Guidelines
  - https://www.jrvs.jp/guideline/ird_rd_guideline.pdf
  - https://johboc.jp/guidebook_2021/doc2-1/
  - https://jams.med.or.jp/guideline/genetics-diagnosis_2022.pdf
  - https://www.ncchd.go.jp/hospital/about/section/iden/dna.html
  - https://jams.med.or.jp/guideline/genetics-diagnosis_qa.html
  - https://johboc.jp/guidebook_g2022/
  - 臨床遺伝専門医制度委員会（2021）臨床遺伝専門医テキスト① 臨床遺伝学総論. 診断と治療社
  - 認定遺伝カウンセラー制度委員会（2023） 遺伝カウンセリング標準テキスト．診断と治療社
  - Wendy R. Uhlmann, Jane L. Schuette, Beverly Yashar（2009）A Guide to Genetic Counseling, Second Edition. Wiley-Blackwel
  - Patricia McCarthy Veach, Bonnie S. LeRoy, Nancy P. Callanan（2018）Facilitating the Genetic Counseling Process. Springer
  - Robert L. Nussbaum, Roderick R. McInnes, Huntington F. Willard（2016）Thompson & Thompson Genetics in Medicine, 8th Edition, Elsevier
